# A case series of Fabry diseases with CKD in Japan

**DOI:** 10.1007/s10157-023-02439-6

**Published:** 2024-01-09

**Authors:** Oi Yusei, Hajime Nagasu, Naoki Nakagawa, Seigo Terawaki, Takahito Moriwaki, Seiji Itano, Seiji Kishi, Tamaki Sasaki, Naoki Kashihara, Takanobu Otomo

**Affiliations:** 1 Department of Health Informatics, Faculty of Health and Welfare Services Administration, Kawasaki University of Medical Welfare, Kurashiki, Okayama Japan; 2 Department of Nephrology and Hypertension, Kawasaki Medical School, 577 Matsushima, Kurashiki, Okayama 701-0192 Japan; 3 Division of Cardiology, Nephrology, Respiratory and Neurology, Department of Internal Medicine, Asahikawa Medical University, Asahikawa, Japan; 4 Department of Molecular and Genetic Medicine, Kawasaki Medical School, 577 Matsushima, Kurashiki, Okayama 701-0192 Japan

**Keywords:** Real-world data, RAS inhibitors, Fabry diseases, SS-MIX2

## Abstract

**Background:**

It is well known that kidney injury is vital organ damage in Fabry disease (FD). Renin–angiotensin system (RAS) inhibitors are known to reduce proteinuria in patients with chronic kidney disease (CKD) by dilating the glomerular export arteries and reducing intraglomerular pressure. This improvement in intraglomerular pressure, although lowering the glomerular filtration rate, is thought to prevent renal damage and be renoprotective in the long term. RAS inhibitors may be effective in FD patients with proteinuria to prevent the progression of kidney disease, however, the degree to which they are used in clinical practice is unknown.

**Methods:**

The J-CKD-DB-Ex is a comprehensive multicenter database that automatically extracts medical data on CKD patients. J-CKD-DB-Ex contains data on 187,398 patients in five medical centers. FD patients were identified by ICD-10. Clinical data and prescriptions of FD patients between January 1 of 2014, and December 31 of 2020 were used for the analysis.

**Results:**

We identified 39 patients with FD from the J-CKD-DB-Ex including those with suspected FD. We confirmed 22 patients as FD. Half of the patients received RAS inhibitors. RAS inhibitors tended to be used in CKD patients with more severe renal impairment.

**Conclusions:**

This case series revealed the actual clinical practice of FD patients with CKD. In particular, we found cases in which patients had proteinuria, but were not treated with RAS inhibitors. The database was shown to be useful in assessing the clinical patterns of patients with rare diseases.

## Introduction

Fabry disease (FD) is an X-linked lysosomal storage disorder that leads to the accumulation of globotriaosylceramide (Gb3) in cells caused by decreased activity of alfa-galactosidase A with pathogenic *GLA* variants [1]. It is well known that kidney injury is a vital organ damage in FD. Pathological changes typically described include vacuolated renal epithelial cells and podocytes in the glomerulus and distal tubules [2]. The incidence of FD worldwide reportedly ranges between 1 in 40,000 and 117,000 in live births for males [3]. On Japanese newborn screening for FD, the frequency of FD patients with pathogenic variants was 1 in 11,854 [4]. Nagata et al*.* reported three FD patients in 2122 male patients with chronic kidney disease (CKD) [5]. The prevalence of biopsy-proven Fabry nephropathy in the Japanese nationwide renal biopsy registry was 0.076% [6]. However, the exact prevalence of FD among patients with renal dysfunction has not been fully explored using large survey.

Currently, enzyme replacement therapy (ERT) to supply alfa-galactosidase intravenously is available and shown effective to be the main treatment for FD patients. There are many studies on the effectiveness of ERT [7–9]. Especially in male patients with FD, ERT treatment is considered essential. Pediatric patients are often treated with ERT once diagnosed, however, some elder patients who were adult-onset or adult-diagnosed FD, do not administer ERT for a variety of reasons. It is unclear whether ERT alone does reduce proteinuria in FD [10].

It is known that renin–angiotensin system (RAS) inhibitors, which are conventionally considered to have a renoprotective effect, mainly involve the correction of intraglomerular pressure. This improvement in intraglomerular pressure can be clinically assessed by the reduction in proteinuria. Therefore, RAS inhibitors may be effective in preventing the progression of renal disease in FD patients with proteinuria.

In FD, guidelines suggest the use of RAS inhibitors in patients with FD with renal complications based on the result of a meta-analysis [11]. However, the degree to which they are used in clinical practice is unknown.

The J-CKD-DB-Ex was constructed in order to clarify the prevalence of various diseases in CKD patients and the actual practices that is a large-scale, nationwide registry based on electronic health record (EHR) data from participating hospitals of university medical schools [12]. To reveal the actual status of FD with CKD patients, we examined using J-CKD-DB-Ex.

## Materials and methods

### Study design and participants

**Fig. 1:**
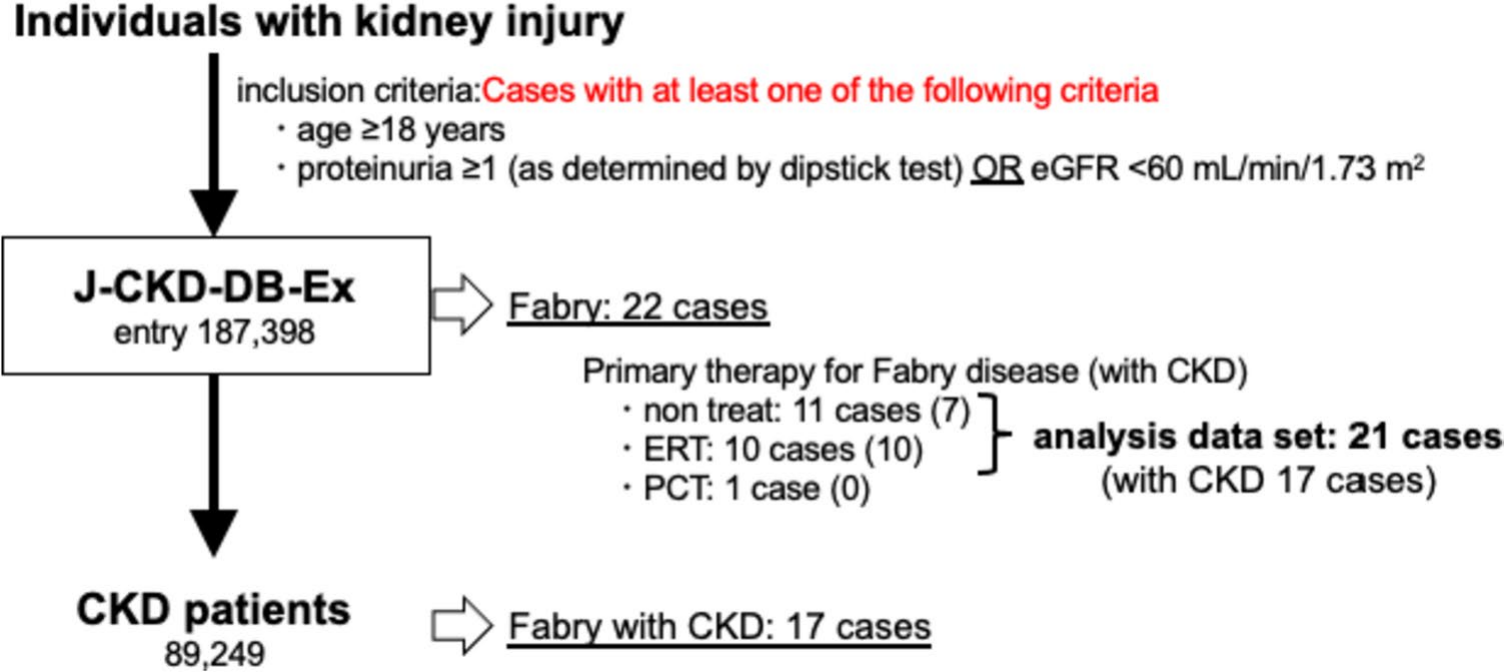
Flowchart of the study population. Of 187,398 patients, 21 were included in this case series. *eGFR* estimated glomerular filtration rate, *CKD* chronic kidney disease, *ERT* enzyme replacement therapy, *PCT* pharmacological chaperone therapy

J-CKD-DB-Ex was developed as a prospective longitudinal CKD database, based on J-CKD-DB system [12, 13]. The J-CKD-DB-Ex study was designed to identify risk factors for estimated glomerular filtration rate (eGFR) decline over time among CKD patients in a real-world practice setting. The inclusion criteria for J-CKD-DB-Ex were individuals aged ≥ 18 years with proteinuria ≥ 1 (as determined by dipstick test) or eGFR < 60 mL/min/1.73 m^2^. The protocol was approved by the Ethics Board of Kawasaki Medical School (No. 5899). The study was performed per relevant guidelines and the Declaration of Helsinki of 1975, as revised in 2013. The data were analyzed anonymously. The study design is shown in Fig. 1. J-CKD-DB-Ex contains data on 187,398 patients with kidney injury between 1 January 2014 and 31 December 2020 from five medical centers. We extracted FD patients identified by ICD-10 from them. All clinical data and prescriptions of FD patients were used for the analysis.

Age, gender, eGFR, creatinine, hemoglobin, serum albumin, total cholesterol (TC), brain natriuretic peptide (BNP), urinary albumin creatinine ratio (ACR), ERT, RAS inhibitor (angiotensin II receptor blockers, angiotensin converting enzyme inhibitors and aldosterone antagonists) usage were extracted to generate a data set.

### Statistical analyses

Baseline statistics are shown as mean ± SD for data with a normal distribution, otherwise as median and interquartile range. Baseline patient characteristics and outcomes were compared using *t* test, or Mann–Whitney *U* test, as appropriate. All statistical analyses were performed using R (version 4.1.2.). Statistical significance was defined as *p* < 0.05, two-tailed.

## Results

### Baseline characteristics

We identified 39 patients with a disease name of “FD” including “suspected FD” from 187,398 entries in the J-CKD-DB-Ex database and 22 patients were confirmed as FD by excluding suspected cases. Additionally, we excluded one patient from our analysis data set who was taking Migalastat hydrochloride from the analysis. Finally, 21 FD with kidney injury patients were analyzed in this study. A profile of the 21 patients is presented (Table 1). The number of males and females was 12:9. The rate of ERT use was also ~ 50%. Biochemical data are presented at the time of the first administration of RAS inhibitors or the first time of data recorded for the RAS inhibitors unused group. The majority of patients also had ACR measurements and demonstrated albuminuria. Half of them were administered with RAS inhibitors. Of these 21 cases, 17 were diagnosed with CKD because of evidence of prolonged status of kidney injury for more than 3 months. Table 2 shows a tabulation of patients according to the use or non-use of RAS inhibitors. Patients with more severe renal dysfunction based on eGFR tended to receive RAS inhibitors.

**Table 1:** Profile of Fabry patients in J-CKD-DB-Ex

Case	age	Sex	ERT	RAS	eGFR	Hb	Alb	TC	BNP	ACR	U-Pro	CKD	OP	HT
1	48	F	1	1	93.2	13.4	4.6	Non	63.4	66.6	(−)	1	1458	0
2	63	F	0	1	55	11.6	3.8	171	85.2	400	(−)	1	161	1
3	46	F	0	1	51.7	11	4.3	212	32.5	Non	(−)	1	289	1
4	36	M	1	1	52.1	14.4	4.5	156	Non	92.3	(−)	1	2541	1
5	56	M	0	1	43.6	16.3	4.8	247	29.7	39.6	(+ −)	1	680	1
6	64	M	0	1	77.2	14.7	4.2	162	251.5	Non	Non	0	1729	1
7	47	M	1	1	26.2	10.9	3.7	140	84.9	374.2	(2 +)	1	743	1
8	80	F	0	1	70	12.7	4	172	Non	200	(−)	1	2373	0
9	41	M	1	1	64	13.7	4	157	87.6	400	(1 +)	1	2480	1
10	58	F	1	1	50.2	13.4	4.1	322	Non	490.5	(1 +)	1	2513	1
11	52	M	0	0	44.6	11.5	4.5	220	90.8	46.9	(−)	1	1031	1
12	43	M	0	0	56.6	13.6	3.9	156	Non	363.2	(+ −)	0	84	1
13	79	M	0	0	54.8	16.1	4	198	184.6	80	(−)	1	198	1
14	38	F	1	0	101.1	13.8	4.5	158	Non	269	(1 +)	1	661	0
15	53	M	1	0	58.2	15	4.1	171	Non	2222.2	(+ −)	1	2493	1
16	51	M	1	0	58.9	15.4	4.3	Non	62.3	200	(+ −)	1	2492	1
17	79	M	0	0	57.9	15.1	3.9	154	887.7	64.8	(−)	0	24	1
18	22	F	0	0	118.3	12.8	4.4	199	Non	Non	(−)	0	2277	0
19	57	F	1	0	49.8	16.6	4.7	329	Non	51.9	(−)	1	2397	0
20	22	M	1	0	128	15.6	4.8	Non	Non	500	(+ −)	1	1826	0
21	68	F	0	0	79	11.7	2.4	Non	172.9	Non	(3 +)	1	126	0

*ERT* enzyme replacement therapy, *RASi* renin–angiotensin system inhibitor, *eGFR* estimated glomerular filtration rate (mL/min/1.73 m^2^), *Hb* hemoglobin (g/dL), *Alb* albumin (g/dL), *TC* total cholesterol (g/dL), *BNP* brain natriuretic peptide (pg/mL), *ACR* urinary albumin creatinine ratio (mg/g/CRN), *U-Pro* proteinuria, *OP* observation period (day), *HT* usage of anti-hypertensive drugs, in the ERT and RAS columns, 1 indicates used and 0 indicates not used; proteinuria was described as a dipstick, *CKD* column, 1 indicates present and 0 indicates absent

**Table 2:** Population of FD patients with and without RAS administration

	Number	Age	Female (number)	ERT (%)	RASi (%)	eGFR	Hb	Alb	TC	BNP	ACR
RASi on	10	53.9 ± 12	5	50	100	58.3 ± 17.7	13.2 ± 1.6	4.2 ± 0.3	193.2 ± 55	90.7 ± 69.5	257.9 ± 167
RASi off	11	51.3 ± 18	4	45	0	73.4 ± 27.8	14.3 ± 1.7	4.1 ± 0.6	198.1 ± 54	279.7 ± 307.6	422.0 ± 654

*ERT* enzyme replacement therapy, *RASi* renin–angiotensin system inhibitor, *eGFR* estimated glomerular filtration rate (mL/min/1.73 m^2^), *Hb* hemoglobin (g/dL), *Alb* albumin (g/dL), *TC* total cholesterol (g/dL), *BNP* brain natriuretic peptide (pg/mL), *ACR* urinary albumin creatinine ratio (mg/g/CRN)

## Discussion

In this case series, we confirmed 22 FD patients with kidney injury. The prevalence estimated from this study is 0.012%, 1 in 8518 among our database entries. The frequency of FD shown here is higher than the overall prevalence of FD in the previous studies. The criteria for inclusion in the J-CKD-DB-Ex are patients with at least one episode of proteinuria or eGFR below 60 (mL/min/1.73 m^2^). The database, therefore, includes proteinuric patients who have not yet developed CKD. Similarly, FD patients who do not show renal dysfunction are not included in the database. In the entire database, 47.6% (89,249/187387) of patients with kidney injury had CKD; whereas 77.3% (17/22) of FD patients had CKD. Thus, it is clear that FD constitutes a major factor in CKD among the diseases causing kidney injury. All patients (10/10) in the ERT group had CKD, while 63.6% (7/11) of the non-ERT group had CKD. Similarly, 90% (9/10) of the patients who received RAS inhibitors had CKD, and 72.7% (8/11) of those who did not receive RAS inhibitors had CKD. These may be due to the fact that FD patients with severe systemic symptoms, including renal impairment, were treated with ERT and RAS inhibitors. However, it is difficult to conclude in this study whether drug therapy is effective in preventing the transition to CKD. Nagata et al*.* diagnosed three cases of FD based on screening of 2122 male CKD patients [5]. It is not easy to determine the exact frequency data for rare diseases such as FD, but it is suggested that FD is more concentrated in patients presenting with renal dysfunction than in the general population. Taken together, the information presented here, the frequency of FD in patients with kidney injury, which was obtained by extracting rare diseases from a database with a large number of entries is considered to be useful.

This study revealed the clinical practice of FD patients with kidney injury. Several meta-analyses and RCTs have reported that RAS inhibitors reduce ESKD progression and all-cause mortality with or without DM complications and regardless of the CKD stage [14–16]. Especially in CKD patients with proteinuria, RAS inhibitors have been shown to significantly improve renal prognosis. In the Fabry outcome survey (FOS) Registry, a high prevalence (57% of male and 47% of female patients) of uncontrolled hypertension was reported in patients with FD which generally increased with worsening CKD stage [17]. These findings suggest that the use of RAS inhibitors is an important treatment option for FD with CKD. It has also been reported that the combination of ERT with a RAS inhibitor significantly reduces proteinuria [10]. Another study showed that RAS inhibitors reduced proteinuria in patients with FD with an improvement of eGFR slopes [18]. However, the results of the present study showed that even FDs with proteinuria did not have adequate RAS inhibitor use. It is unclear whether this practice pattern is due to the lack of evidence for RAS inhibitors in patients with FD. Alternatively, it may be due to problems with health insurance coverage of the drugs or the diversity of specialties treating patients with FD. Furthermore, in this study, there was a trend towards lower eGFR on the index day (i.e., the day the RAS inhibitor was started) in the RAS inhibitor administrated group (Table 2). The possible explanation for this is that the administration of RAS inhibitors was considered when renal failure progressed in FD, the same discussion as above. Early initiation of RAS inhibitors in FD patients with proteinuria may preserve renal function in the long term, although more evidence needs to be accumulated.

The present study has several limitations. First, the small sample size was still insufficient for a rare disease, FD, to draw definite conclusions from this study. Indeed it was unable to analyze the eGFR slope because only a few patients had data for the period appropriate for the analysis. Second, data on genetic information, family history, symptoms, and blood pressure levels are not available in the J-CKD-DB-Ex. Another weakness of this database is that it only collects data from university hospitals. However, FD is an intractable disease and its diagnosis is not easy. Therefore, we believe that many FD patients are likely to be diagnosed and managed at a highly specialized hospital such as a university hospital. We hope to further validate the data with an expanded database in the future. Many FD patients were on other anti-hypertensive drugs. This data shows a background of patients receiving RASi in accordance with hypertension. This may be characteristic of adult FD. Furthermore, there is no information on genomic mutation in this study. There is no information on genomic information in this database. Genetic mutations such as E66Q, S126G, and D313Y may affect the indication for treatment of ERT [19]. The frequency of such mutations is also not high, so the results of this study are not expected to have a strong impact. Therefore, some research questions related to these variables were not answered. In addition, this database only includes patients who showed renal dysfunction. Since FD patients who have never shown kidney injury are not included in the database, it is not possible to examine the impact of RAS inhibitors on the incidence of CKD.

This case series provided information on a certain number of FD patients and revealed the actual situation of clinical practice of FD patients with kidney injury. We found cases in which RAS inhibitors were not administered even to patients with proteinuria, and we believe that establishing evidence and informing the public may contribute to improving renal prognosis by increasing the compliance rate. The database was shown to be useful in assessing the clinical patterns of patients with rare diseases.

## Data Availability

The datasets were generated at Kawasaki Medical School. Derived data supporting the findings of this study are available from the corresponding author on reasonable request.
